# Association Between Common Systemic Medications and the Presence and Severity of Furcation Involvement: A Cross-Sectional Study

**DOI:** 10.3390/healthcare13222930

**Published:** 2025-11-16

**Authors:** Georgios S. Chatzopoulos, Larry F. Wolff

**Affiliations:** 1 Division of Periodontology, Department of Developmental and Surgical Sciences, School of Dentistry, University of Minnesota, Minneapolis, MN 55455, USA; wolff001@umn.edu; 2 Department of Preventive Dentistry, Periodontology and Implant Biology, School of Dentistry, Aristotle University of Thessaloniki, 54124 Thessaloniki, Greece

**Keywords:** periodontitis, furcation defects, medications, cardiovascular drugs, risk

## Abstract

Background/Objectives: While furcation involvement is a known predictor for tooth loss, the role of systemic medications is understudied. This study aimed to investigate the association between common systemic medications and both the presence and severity of furcation involvement in a large patient cohort. Methods: This retrospective cross-sectional study analyzed electronic health records from 15,881 patients within the BigMouth Dental Data Repository. The association between demographics, medication use (ACE inhibitors, statins, anti-coagulants, antidepressants, bisphosphonates, proton pump inhibitors), and the presence of furcation involvement was assessed using Chi-Square tests and multivariate logistic regression. The statistically significant relationship between medications and furcation severity (Grades 1–4) was analyzed using multinomial logistic regression. Results: Being male (OR: 1.34) and of non-Hispanic ethnicity (OR: 1.36) were significant demographic predictors for furcation involvement. After adjusting for demographics, use of ACE inhibitors (OR: 1.40), anti-coagulants (OR: 1.19), and statins (OR: 1.14) were significantly associated with higher odds of furcation involvement. Specifically, Lisinopril (OR: 1.48), Enalapril (OR: 1.83), and Atorvastatin (OR: 1.27) were significant predictors. Furthermore, patients taking Lisinopril, Aspirin, Atorvastatin, or Simvastatin had approximately 1.5 times the odds of having Grade 3 involvement compared to Grade 1 (*p* ≤ 0.001). Conclusions: The use of certain systemic medications, particularly for cardiovascular conditions, is independently associated with both a higher likelihood and increased severity of furcation involvement, highlighting the critical need for dental professionals to consider a patient’s medication profile as an integral part of periodontal risk assessment.

## 1. Introduction

Periodontitis is a chronic inflammatory condition featured by a gradual destruction of periodontal structures, including connective tissue attachment and alveolar bone [1]. Staging and grading are based on the severity of disease, complexity of management, and pace of progression [1]. The etiology of periodontitis entails a dysbiotic dental biofilm, representing an imbalance in the microbial community that initiates an inappropriate host immune response and thereby the destruction of the periodontal tissues [2,3]. Untreated periodontitis is associated with a high risk of tooth loss, which can lead to severe limitations of oral function, aesthetic appearance, and general well-being of the individual [4,5]. Considering global epidemiologic information, according to the Global Burden of Disease Study, the number of people suffering from severe periodontitis worldwide is approximately 1.1 billion, and it causes a considerable economic burden for national healthcare [6,7].

The presence of bone resorption in the bifurcation and trifurcation of multirooted teeth is referred to as furcation involvement [8]. When the furcation surfaces are exposed by periodontal destruction, however, these sites are then well-suited for the growth of periodontopathic bacteria. This microbial adherence further provides an increased hostile milieu for standard plaque control measures and root surface debridement [9]. A meta-analysis by Nibali and coworkers [10] evaluated the risks of tooth loss in molars with furcation involvement. This review included 21 longitudinal studies that had a minimum three-year follow-up. Combined, these findings indicated that, while under supportive periodontal treatment for up to 10 to 15 years, molars with furcation involvement had about twice the risk of being lost. An increased risk of tooth loss was also reported by the review; the more severe the furcation involvement, the more likely tooth loss, with extreme and severe furcations posing the greatest risk to tooth retention.

A comprehensive meta-analysis conducted by Helal et al. [11] strongly supported furcation involvement as a significant predictor of tooth loss. This large-scale, pooling data meta-analysis offered powerful evidence of a pronounced association between furcation defects and tooth loss in periodontitis patients. Apart from furcation involvement, the meta-analysis uncovered other important contributors to tooth loss. These features were advanced patient age, which was associated with an odds ratio of 1.020 per year (95% confidence interval, 1.015 to 1.025), and the behavior habit of smoking, which was associated with an odds ratio of 1.305 (95% confidence interval, 1.124 to 1.515). The cumulative nature of these findings, which are present in multiple independent populations, as critically presented in this meta-analysis, confirms that furcation status is a central determinant for retaining teeth in the presence of periodontitis over the long term.

The expanding body of evidence connecting periodontal disease to numerous systemic conditions has led to the establishment of “periodontal medicine,” a field dedicated to studying the relationship between oral and overall health [12]. Research has identified links between periodontal disease and a multitude of systemic disorders, with prominent examples including diabetes, cardiovascular disease, obesity, preterm birth, cancer, osteoporosis, and various other conditions such as COPD, HIV, and thyroid disorders [13,14]. Significantly, periodontitis has been shown to be independently associated with most chronic noncommunicable diseases of aging and with premature mortality [15]. Discrepancies in the reported findings on these systemic links may be due to variations in the severity and level of control of the systemic diseases among the patient populations studied. The use of certain medications, which can reflect the severity of a systemic condition, might also explain some of the observed associations with periodontitis.

While the existing literature firmly establishes furcation involvement as a critical predictor for tooth loss and acknowledges the broad connections between periodontitis and numerous systemic diseases, a specific gap remains. The current understanding often attributes variations in the periodontitis–systemic disease link to the severity of the systemic condition itself, but it largely overlooks the direct role that the medications used to manage these conditions might play. Since the use of certain drugs can be an indicator of disease severity and can have its own biological effects, it is crucial to disentangle the impact of the medication from the disease itself. There is a lack of large-scale studies that specifically investigate whether common systemic medications are independently associated with the presence and, importantly, the severity of furcation involvement.

Drug–periodontal interactions are biologically plausible due to the shared inflammatory and bone metabolic pathways involved in both drug action and periodontal disease pathogenesis. Angiotensin-converting enzyme (ACE) inhibitors may modulate local immune responses and vascularity, but Mendelian randomization and observational data suggest that ACE inhibitors are associated with an increased risk of acute periodontitis. This increased risk might occur via effects on host immune cell infiltration and pro-inflammatory signaling, including dendritic cell and osteoclast activation [16,17,18]. Conversely, Statins exhibit pleiotropic effects beyond lipid-lowering, including anti-inflammatory actions, inhibition of osteoclastogenesis, and promotion of osteoblast activity. These effects can reduce alveolar bone loss and periodontal inflammation by modulating cytokine profiles and bone morphogenetic protein expression [19,20,21]. Clinical and preclinical studies demonstrate that statins can dampen inflammatory mediators and preserve bone in periodontal tissues.

Therefore, the primary aim of this study was to investigate the association between the use of common systemic medications and both the presence and severity of furcation involvement within a large patient cohort. The study hypothesized that the use of specific medication classes, particularly those for cardiovascular conditions such as ACE inhibitors, statins, and anti-coagulants, would be associated with significantly higher odds of having furcation involvement (primary hypothesis). A secondary hypothesis was that the use of these medications would also be positively correlated with an increased grade of furcation involvement, suggesting a potential link between these drugs and the progression of periodontal destruction in multi-rooted teeth (secondary hypothesis).

## 2. Materials and Methods

### 2.1. Study Design and Data Collection

This cross-sectional, retrospective study received a determination from the University of Minnesota Institutional Review Board (STUDY00016576) that it did not constitute research involving human subjects, as defined by the Department of Health and Human Services and the United States Food and Drug Administration. Further approval was granted by the BigMouth Consortium for Oral Health Research and Informatics clinical review committee. The study adhered to the Helsinki Declaration of 1975, as most recently revised in 2013.

### 2.2. Data Source and Patient Selection

Electronic health records (EHRs) from 2011 to 2021 were extracted from the BigMouth Dental Data Repository. This repository contains data from university dental clinics affiliated with the BigMouth network, specifically, Harvard University, University of Texas Health, University of California, San Francisco, University of Colorado, Loma Linda University, University of Buffalo, The University of Iowa, and The University of Minnesota. The dental charts of adult (≥18 years) patients who sought dental therapy at these clinics and consented to the study protocol were evaluated. EHRs were completed by dental students, residents, and faculty oral healthcare providers during patient visits, utilizing Dental Procedure Codes and Current Procedural Terminology (CPT) procedures. The Current Dental Terminology (CDT), a code set developed and updated by the American Dental Association (ADA) for reporting dental services, was specifically used.

Patients were included if they had at least one completed treatment code for a comprehensive oral evaluation (D0150), a periodic oral evaluation (D0120), or a comprehensive restorative and periodontal exam (D0180). Furthermore, patients identified as having periodontitis were included if their records contained any of the following CDT codes indicating non-surgical or surgical periodontal treatment: D4210, D4211, D4240, D4241, D4245, D4260, D4261, D4263, D4266, D4274, D4341, D4342, or D4910.

### 2.3. Data Extraction and Variables

Clinical data pertaining to furcation involvements of molar teeth, completed by dental students, residents, and faculty members from the participating institutions, were extracted. The presence and degree of furcation involvement were assessed and recorded according to standardized clinical protocols and diagnostic codes utilized by the BigMouth Consortium-affiliated institutions. In cases of multiple examinations for the same patient, only the initial assessment was included in the analysis. Clinical data detailing the presence and degree of furcation involvement were extracted for all types of molar teeth, specifically including first, second, and third maxillary molars, and first, second, and third mandibular molars. This comprehensive approach allowed for a thorough analysis of the occurrence, severity, and precise distribution of these defects across the various molar types. Relevant data extracted from patient EHRs included demographic characteristics, dental procedural codes, and self-reported medications for all patients meeting the inclusion criteria.

### 2.4. Independent Variables

The following independent variables were included:

Demographic characteristics: age (at the time of CDT code completion), ethnicity, race, gender, smoking habits, and self-reported alcohol consumption.

Patient-reported medication intake including ACE inhibitors (Benazepril, Enalapril, Lisinopril, Quinapril, Ramipril); antidepressants (fluoxetine, paroxetine, sertraline, citalopram, duloxetine, venlafaxine, desvenlafaxine, trazodone, mirtazapine, bupropion, nortriptyline, doxepin, quetiapine, aripiprazole, lithium, Amitryptiline); anti-coagulants (Apixaban, Plavix, Xarelto, Brilinta, Cilostazol, Aspirin, Clopidogrel, Warfarin); statins (Atorvastatin, Lovastatin, Pravastatin, Rosuvastatin, Simvastatin); bisphosphonates (Zoledronic acid, ibandronate, alendronate, risedronate); and proton pump inhibitors (Omeprazole, Lansoprazole, Dexilant, Esomeprazole, Pantoprazole).

### 2.5. Statistical Analysis

To investigate the relationship between patient characteristics and furcation involvement, a multi-step statistical analysis was conducted. Initially, to determine if there was an association between various factors (such as gender, race, and medication use) and the simple presence or absence of any furcation involvement, Chi-Square and Fisher’s Exact tests were employed. These tests identified which variables had a statistically significant relationship with having the condition. Following this, for the cohort of patients with furcation involvement, the distribution across the different levels of severity—Grades 1, 2, 3, and 4—was analyzed using Chi-Square tests to see if medication use was associated with a different pattern of severity. Finally, to understand the independent impact of each significant factor while controlling for others, two types of regression analyses were performed. A multivariate logistic regression was used to calculate the odds ratios (ORs) for having any furcation involvement, providing insight into the strength of association for each variable, while controlling for the demographic variables (age, gender, ethnicity, race, smoking habits, and self-reported alcohol consumption). To further explore the effect on severity, a multinomial logistic regression was performed to determine how medication use affected the odds of a patient presenting with a higher grade of furcation (e.g., Grade 2 or 3) compared to the baseline of Grade 1. Model fit indicators were used to assess model quality and confirmed an adequate fit.

## 3. Results

**Figure 1:**
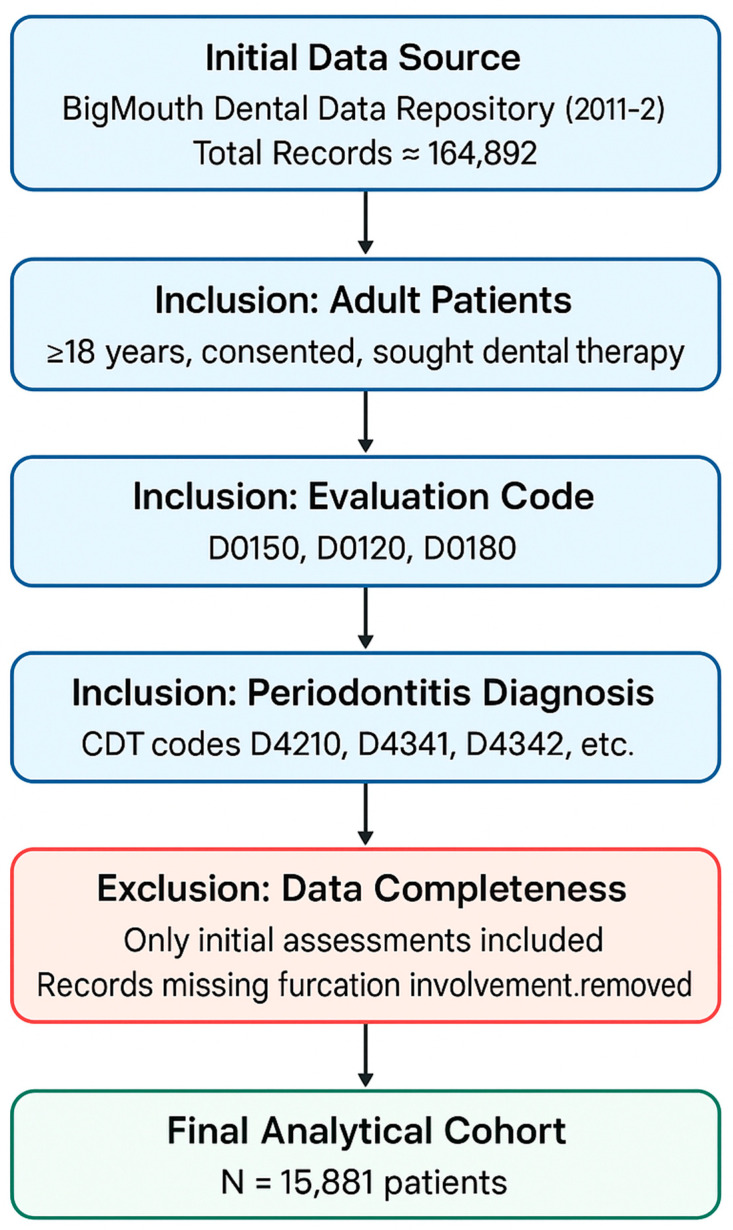
Flow diagram of patient selection for the cross-sectional analysis.

An initial statistical analysis of the 15,881-patient cohort identified significant associations between furcation involvement and several key factors. The large sample size made it possible to conduct a strong statistical analysis. The flow diagram of patient selection for the cross-sectional analysis is shown in Figure 1. Statistically significant differences were found for demographic categories, including gender, race, and ethnicity (*p* < 0.0001 for all). Notably, male patients (69.8%) and White patients (69.4%) demonstrated a higher prevalence of furcation involvement compared to their counterparts. The use of broad medication classes, including ACE inhibitors, anti-coagulants, and statins, was also significantly associated with a higher frequency of furcation involvement (*p* < 0.0001 for all). When this analysis was extended to specific medications, numerous individual drugs within these and other categories were also found to have a statistically significant association with the presence of furcation involvement.

The statistical association of specific medications with furcation involvement is shown in Table 1. ACE inhibitors and statins showed a statistically significant association (*p* < 0.05), whereas none of the listed antidepressants or proton pump inhibitors had a significant relationship with furcation involvement. Furthermore, a significant association was found for numerous anti-coagulants, including Aspirin (*p* < 0.0001), Clopidogrel (*p* < 0.0001), Warfarin (*p* = 0.0270), Plavix (*p* = 0.0132), Xarelto (*p* = 0.0382), and Apixaban (*p* = 0.0469). Within the bisphosphonate class, the association was limited, with only Alendronate use being statistically significant (*p* = 0.0478).

To assess the independent impact of these factors, multivariate logistic regression analyses were conducted. After controlling for significant demographic variables, an analysis focusing on general medication classes revealed that patients taking ACE inhibitors (OR: 1.40, *p* < 0.0001), anti-coagulants (OR: 1.19, *p* = 0.0150), and statins (OR: 1.14, *p* = 0.0399) had significantly greater odds of presenting with furcation involvement. A separate, more detailed regression model that included specific medications further clarified these findings. The adjusted model controlled for key demographic factors, including age, gender, ethnicity, race, smoking habits, and self-reported alcohol consumption. The multivariate logistic regression analysis of specific medications and demographics as predictors for furcation involvement is shown in Table 2. In this model, demographic factors such as being male (OR: 1.34, *p* < 0.0001) and of non-Hispanic ethnicity (OR: 1.36, *p* = 0.0001) remained significant predictors. The use of specific medications like Lisinopril (OR: 1.48, *p* < 0.0001), Enalapril (OR: 1.83, *p* = 0.0179), and Atorvastatin (OR: 1.27, *p* = 0.0051) was also confirmed to be associated with significantly higher odds of furcation involvement.

An initial analysis was performed to see if patients taking specific medications were distributed differently across the four grades of furcation involvement. The patient distribution by specific medication and furcation grade is shown in Table 3. Using a Chi-Square test, the study found that for most medications, including all listed antidepressants and bisphosphonates, the distribution was not statistically significant (*p* > 0.05). However, a significant, non-random distribution across the grades was identified for patients taking four specific drugs: Lisinopril (*p* = 0.0000), Aspirin (*p* = 0.0000), Atorvastatin (*p* = 0.0001), and Simvastatin (*p* = 0.0435).

A more advanced multinomial logistic regression analysis was then used on these four significant medications to determine the odds of a patient being in a higher furcation grade compared to Grade 1, as demonstrated in Table 4. For the Grade 2 versus Grade 1 comparison, only taking Lisinopril was associated with significantly higher odds (OR: 1.15, *p* = 0.038). The most substantial finding was in the Grade 3 versus Grade 1 comparison, where all four medications were associated with significantly higher odds. Patients taking these drugs had approximately 1.5 times the odds of having Grade 3 furcation involvement compared to Grade 1, with odds ratios of 1.50 for Lisinopril, 1.48 for Aspirin, 1.54 for Atorvastatin, and 1.46 for Simvastatin (*p* ≤ 0.001 for all).

**Figure 2:**
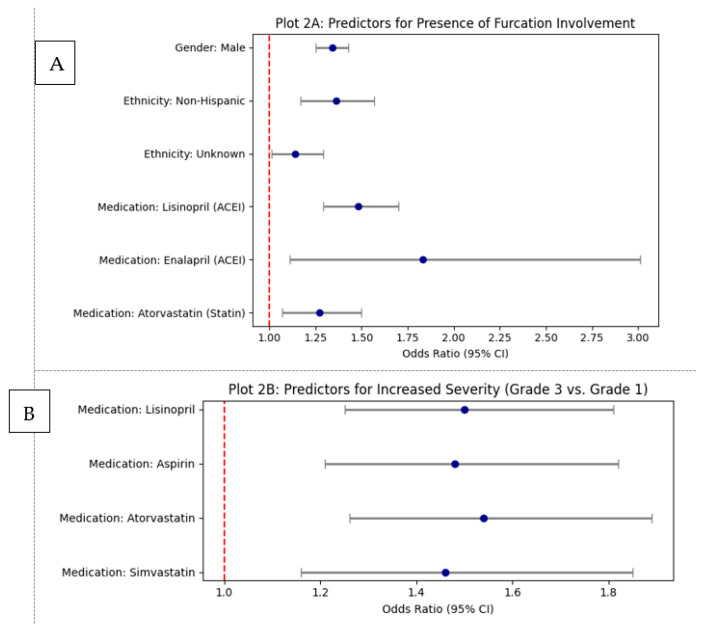
Graphical summary of significant odds ratios for furcation involvement: (**A**) Predictors for the presence of any furcation involvement (multivariate logistic regression). (**B**) Predictors for increased furcation severity (Grade 3 vs. Grade 1 multinomial logistic regression).

Finally, the analysis investigated the relationship between these medications and the most severe category, Grade 4. The results showed no statistically significant (*p* > 0.05) association between taking any of the four drugs and the odds of having Grade 4 furcation involvement compared to Grade 1. This lack of significance is likely due to the very small number of patients within the Grade 4 category, which may have been insufficient for a meaningful statistical comparison. Therefore, while these medications are linked to higher odds of moderate furcation disease, the association does not extend to the most severe grade in this cohort. The graphical summary of significant odds ratios for furcation involvement is shown in Figure 2.

## 4. Discussion

The present study investigated the relationship between the use of common systemic medications and both the presence and severity of furcation involvement in a large patient cohort. To the best of our knowledge, this is the first study with such a large sample size (15,881 cases from a pool of 164,892 records) and an extensive attempt to explore patient factors associated with the presence of furcation involvement in a multi-institutional EHR analysis of periodontitis patients. The large sample size made it possible to conduct a strong statistical analysis. Furcation involvement, a condition commonly associated with the progression of periodontal disease, can be influenced not only by local oral factors but also by systemic medications. Certain medications—such as anti-coagulants, statins, and ACE inhibitors—have been linked to a higher risk of periodontitis, which may in turn exacerbate furcation defects [22]. Additional medications, including antidepressants, oral hypoglycemic agents, calcium channel blockers, insulin, diuretics, and anticonvulsants, have also been implicated in increasing the likelihood or severity of periodontitis in various studies [18]. These associations highlight the importance of considering patients’ systemic health and medication history in periodontal diagnosis and management.

On the other hand, some medications have shown potential benefits in the treatment of furcation involvement, particularly when applied locally in combination with scaling and root planing [23]. Medications such as alendronate, rosuvastatin, and simvastatin have demonstrated both clinical and radiographic improvements in furcation defects. Boric acid has also been found to enhance bone healing and reduce defect depth when used adjunctively with non-surgical periodontal treatment [23]. Moreover, tetracycline has shown some promise in improving furcation outcomes, although its effects may be limited to shorter durations. These findings suggest that certain pharmacological agents, when used strategically, may offer therapeutic support in managing complex periodontal conditions [23].

Analyzing electronic health records from 15,881 patients, significant associations were found between certain medication classes and furcation involvement. Specifically, the use of ACE inhibitors, anti-coagulants, and statins was significantly linked to higher odds of furcation involvement. Individual drugs like Lisinopril, Enalapril, and Atorvastatin also showed strong associations. A key finding of the study is the independent association of these medications, particularly those used for cardiovascular conditions, with both the likelihood and increased risk for severity of furcation involvement. For instance, patients taking Lisinopril, Aspirin, Atorvastatin, or Simvastatin had approximately 1.5 times the odds of having Grade 3 furcation involvement compared to Grade 1. This suggests that these medications may not only be indicators of underlying systemic conditions that predispose individuals to periodontitis but might also have a direct or indirect role in the progression of periodontal destruction within the furcation areas of multi-rooted teeth.

Systemic medications impacting inflammation, bone metabolism, or vascularity have the potential to influence periodontal outcomes by modifying the host’s response to bacterial challenges, altering the balance of bone resorption, and affecting tissue repair. For instance, pharmaceuticals that suppress inflammatory processes (such as statins or DMARDs) or inhibit osteoclast function (like bisphosphonates) might mitigate the destruction of periodontal tissues. Conversely, agents that negatively affect vascularity (e.g., anti-VEGF therapies) could hinder healing and elevate disease risk [19,20,21,24]. However, when analyzing these relationships, it is vital to account for confounding variables like underlying systemic conditions (such as diabetes or cardiovascular disease), smoking habits, and advanced age, as these factors independently raise the risk of periodontitis and often necessitate medication use [18,25,26]. Clinically, understanding these complex drug–disease interactions is essential for risk stratification and ensuring optimal periodontal management, particularly in patients on these therapies, where adjunctive pharmacologic modulation might improve outcomes but requires meticulous consideration of systemic comorbidities and potential drug-specific adverse effects.

The study highlights a crucial gap in existing literature, which often attributes variations in the periodontitis–systemic disease link solely to the severity of the systemic condition, overlooking the direct impact of the medications themselves. While the study found significant associations with moderate (Grade 3) furcation involvement, it did not find a statistically significant association with the most severe furcation defect (Grade 4), likely due to the small number of patients in that category. The findings reveal that patients using certain cardiovascular drugs face approximately 1.5 times the odds of having Grade 3 furcation involvement compared to Grade 1. This quantitative association underscores the critical importance of considering a patient’s complete medication profile during periodontal risk assessment, as it could offer valuable insights into their susceptibility and the potential progression of furcation defects, guiding the implementation of more aggressive preventive or supportive periodontal therapy.

Findings in the current study have limitations related to its retrospective, cross-sectional nature, despite being strengthened by large numbers of patients and the multi-institutional database. Because of the use of electronic health records, data completeness is reliant on complete and consistent documentation by multiple dental students, residents, and faculty. This might also result in variation in the recording of furcation involvement, hence inaccuracies in our analyses of severity and distribution. A subtle bias for generalization may persist due to missing data, since records that contained no information on furcation involvement were eliminated. Additionally, because of the cross-sectional design, we were unable to show a causal relation between the factors found and the involvement of furcation defect; therefore, it can only be said that factors are associated with furcation involvement. There may be other potential confounders not present in EHRs, such as oral hygiene habits, specific bacterial profiles, or a patient’s adherence to treatment, that might also affect the apparent relationships. Future prospective studies or clinical trials are necessary to verify the causal nature of these associations.

Our findings offer a cautious but persuasive interpretation of the dynamics of furcation involvement. This study holds significant importance in several key areas. Firstly, it addresses a specific gap in the current understanding of periodontal disease and its systemic links. While it is well-established that furcation involvement is a critical predictor for tooth loss and that periodontitis is broadly connected to numerous systemic diseases, the literature often attributes variations in this periodontitis–systemic link solely to the severity of the systemic condition, largely overlooking the direct role of the medications used to manage these conditions. This research specifically investigates whether common systemic medications are independently associated with the presence and severity of furcation involvement. Secondly, the findings highlight the practical importance of considering patient medication profiles in periodontal risk assessment. By identifying that the use of certain systemic medications, particularly for cardiovascular conditions, is independently associated with both a higher likelihood and increased severity of furcation involvement, the study provides clinicians with crucial information. This can lead to more comprehensive patient evaluations, potentially influencing treatment planning and preventive strategies for individuals on these medications, ultimately aiming to improve long-term tooth retention.

The multi-institutional component of the BigMouth Dental Data Repository increases the generalizability of our results. It is the use of data from eight geographically varied university dental schools within the US that helps make the study sample more representative than it would have been had it involved only one institution. This allows our findings to be more generalizable to adult patients who present for dental disease treatment, especially those with periodontitis, in a dental school-related clinic. Nevertheless, external validity to the wider general population may be restricted, since university clinic attendees are likely to have a unique set of needs and may be more complicated and advanced. While the use of academic data may introduce minor variations due to differing clinical protocols between university and private practice settings, the inclusion of a large sample size and comprehensive data collection process enhanced the external validity of our findings. Consequently, these results should be considered by public health initiatives when developing clinical guidelines for managing furcation-involved molars.

## 5. Conclusions

In conclusion, this study establishes a significant and independent association between the use of specific systemic medications, particularly those for cardiovascular conditions like ACE inhibitors, anti-coagulants, and statins, and both the presence and increased severity of furcation involvement. These findings highlight the critical need for dental professionals to consider a patient’s medication profile as an integral part of periodontal risk assessment. By integrating medication status into risk assessment, clinicians can gain a more comprehensive understanding of a patient’s susceptibility to and progression of furcation defects, ultimately informing individualized, risk-based periodontal management strategies designed to improve long-term tooth retention and include strong preventive measures and interdisciplinary collaboration with other healthcare providers.

## Figures and Tables

**Table 1:** Statistical association of specific medications with furcation involvement.

Medication Class	Specific Medication	Patients with Furcation Involvement (N)	With Furcation (%)	Patients without Furcation Involvement (N)	Without Furcation (%)	Total Patients (N)	*p*-Value
**ACE Inhibitors**	Lisinopril	661	72.8%	247	27.2%	908	<0.0001
	Benazepril	83	77.6%	24	22.4%	107	0.0006
	Ramipril	56	77.8%	16	22.2%	72	0.0008
	Enalapril	52	78.8%	14	21.2%	66	0.0004
	Quinapril	32	84.2%	6	15.8%	38	0.0001
**Antidepressants**	Trazodone	145	67.4%	70	32.6%	215	0.8143
	Sertraline	102	68.0%	48	32.0%	150	0.6953
	Citalopram	96	68.1%	45	31.9%	141	0.7029
	Fluoxetine	92	64.3%	51	35.7%	143	0.4497
	Bupropion	86	65.6%	45	34.4%	131	0.5471
	Venlafaxine	69	66.3%	35	33.7%	104	0.8576
	Duloxetine	57	69.5%	25	30.5%	82	0.5592
	Mirtazapine	53	69.7%	23	30.3%	76	0.5891
	Amitriptyline	49	71.0%	20	29.0%	69	0.4192
	Paroxetine	47	66.2%	24	33.8%	71	0.9413
	Quetiapine	28	65.1%	15	34.9%	43	0.8804
	Nortriptyline	18	72.0%	7	28.0%	25	0.4900
	Desvenlafaxine	10	71.4%	4	28.6%	14	0.6482
	Aripiprazole	7	63.6%	4	36.4%	11	1.0000
	Doxepin	3	75.0%	1	25.0%	4	0.5960
	Lithium	2	66.7%	1	33.3%	3	1.0000
**Anti-coagulants**	Aspirin	549	71.0%	224	29.0%	773	0.0000
	Clopidogrel	163	73.1%	60	26.9%	223	0.0000
	Warfarin	90	71.4%	36	28.6%	126	0.0270
	Plavix	62	72.9%	23	27.1%	85	0.0132
	Xarelto	47	72.3%	18	27.7%	65	0.0382
	Apixaban	30	73.2%	11	26.8%	41	0.0469
	Brilinta	3	75.0%	1	25.0%	4	0.5960
	Cilostazol	2	100.0%	0	0.0%	2	0.5484
**Statins**	Atorvastatin	425	70.0%	182	30.0%	607	0.0001
	Simvastatin	258	69.9%	111	30.1%	369	0.0011
	Pravastatin	119	70.8%	49	29.2%	168	0.0076
	Lovastatin	91	70.0%	39	30.0%	130	0.0264
	Rosuvastatin	82	70.7%	34	29.3%	116	0.0280
**Bisphosphonates**	Alendronate	79	72.5%	30	27.5%	109	0.0478
	Risedronate	5	71.4%	2	28.6%	7	0.6558
	Ibandronate	4	66.7%	2	33.3%	6	1.0000
	Zoledronic acid	1	100.0%	0	0.0%	1	1.0000
**Proton Pump Inhibitors**	Omeprazole	214	67.5%	103	32.5%	317	0.8354
	Pantoprazole	133	66.8%	66	33.2%	199	0.9423
	Lansoprazole	57	69.5%	25	30.5%	82	0.5592
	Esomeprazole	42	67.7%	20	32.3%	62	0.8374
	Dexilant	2	66.7%	1	33.3%	3	1.0000

**Table 2:** Multivariate logistic regression analysis of specific medications and demographics as predictors for furcation involvement.

Parameter	Category	Odds Ratio (OR)	95% CI Lower	95% CI Upper	*p*-Value
**Medication**	Takes_Lisinopril	1.48	1.29	1.70	0.0000
	Takes_Benazepril	0.72	0.45	1.14	0.1601
	Takes_Ramipril	2.21	0.97	5.04	0.0588
	Takes_Enalapril	1.83	1.11	3.01	0.0179
	Takes_Quinapril	1.46	0.53	4.07	0.4687
	Takes_Aspirin	1.14	0.97	1.33	0.1064
	Takes_Clopidogrel	0.79	0.55	1.13	0.1993
	Takes_Warfarin	1.63	0.88	3.04	0.1221
	Takes_Plavix	1.41	0.88	2.25	0.1508
	Takes_Xarelto	1.28	0.71	2.33	0.4124
	Takes_Apixaban	2.65	0.58	12.21	0.2104
	Takes_Atorvastatin	1.27	1.07	1.50	0.0051
	Takes_Simvastatin	1.00	0.81	1.22	0.9634
	Takes_Pravastatin	1.16	0.86	1.58	0.3308
	Takes_Lovastatin	1.06	0.66	1.71	0.8034
	Takes_Rosuvastatin	0.86	0.58	1.28	0.4532
	Takes_Alendronate	1.35	0.89	2.05	0.1528
**Demographics**	Gender: Male	1.34	1.25	1.43	0.0000
	Ethnicity: Non-Hispanic	1.36	1.17	1.57	0.0001
	Ethnicity: Unknown	1.14	1.01	1.29	0.0406
	Race: Black or African American	1.12	0.57	2.19	0.7455

**Table 3:** Patient distribution by specific medication and furcation grade.

Medication Class	Specific Medication	Grade 1 (N)	Grade 1 (%)	Grade 2 (N)	Grade 2 (%)	Grade 3 (N)	Grade 3 (%)	Grade 4 (N)	Grade 4 (%)	Total Patients (N)	*p*-Value
**ACE Inhibitors**	Lisinopril	661	41.9%	722	45.8%	194	12.3%	1	0.1%	1578	0.0000
	Benazepril	83	43.7%	89	46.8%	18	9.5%	0	0.0%	190	0.6548
	Ramipril	56	44.1%	58	45.7%	13	10.2%	0	0.0%	127	0.8227
	Enalapril	52	43.3%	55	45.8%	13	10.8%	0	0.0%	120	0.8441
	Quinapril	32	43.2%	35	47.3%	7	9.5%	0	0.0%	74	0.8651
**Antidepressants**	Trazodone	145	42.5%	158	46.3%	38	11.1%	0	0.0%	341	0.6865
	Sertraline	102	42.3%	109	45.2%	30	12.4%	0	0.0%	241	0.9103
	Citalopram	96	42.3%	104	45.8%	27	11.9%	0	0.0%	227	0.8624
	Fluoxetine	92	41.8%	101	45.9%	27	12.3%	0	0.0%	220	0.8906
	Bupropion	86	42.4%	91	44.8%	26	12.8%	0	0.0%	203	0.9702
	Venlafaxine	69	42.1%	75	45.7%	20	12.2%	0	0.0%	164	0.9419
	Duloxetine	57	42.5%	61	45.5%	16	11.9%	0	0.0%	134	0.9254
	Mirtazapine	53	42.4%	57	45.6%	15	12.0%	0	0.0%	125	0.9427
	Amitriptyline	49	43.0%	52	45.6%	13	11.4%	0	0.0%	114	0.8967
	Paroxetine	47	42.3%	51	45.9%	13	11.7%	0	0.0%	111	0.8953
	Quetiapine	28	43.1%	30	46.2%	7	10.8%	0	0.0%	65	0.9056
	Nortriptyline	18	42.9%	20	47.6%	4	9.5%	0	0.0%	42	0.9213
	Desvenlafaxine	10	41.7%	11	45.8%	3	12.5%	0	0.0%	24	0.9754
	Aripiprazole	7	41.2%	8	47.1%	2	11.8%	0	0.0%	17	0.9760
	Doxepin	3	42.9%	3	42.9%	1	14.3%	0	0.0%	7	0.9892
	Lithium	2	40.0%	2	40.0%	1	20.0%	0	0.0%	5	0.9818
**Anti-coagulants**	Aspirin	549	41.9%	591	45.1%	169	12.9%	2	0.2%	1311	0.0000
	Clopidogrel	163	42.0%	178	45.9%	47	12.1%	0	0.0%	388	0.2520
	Warfarin	90	42.7%	96	45.5%	25	11.8%	0	0.0%	211	0.7788
	Plavix	62	42.5%	68	46.6%	16	11.0%	0	0.0%	146	0.6865
	Xarelto	47	41.6%	52	46.0%	14	12.4%	0	0.0%	113	0.8711
	Apixaban	30	42.3%	33	46.5%	8	11.3%	0	0.0%	71	0.8651
	Brilinta	3	42.9%	3	42.9%	1	14.3%	0	0.0%	7	0.9892
	Cilostazol	2	50.0%	1	25.0%	1	25.0%	0	0.0%	4	0.8944
**Statins**	Atorvastatin	425	41.8%	463	45.5%	129	12.7%	0	0.0%	1017	0.0001
	Simvastatin	258	42.1%	277	45.2%	78	12.7%	0	0.0%	613	0.0435
	Pravastatin	119	42.2%	129	45.7%	34	12.1%	0	0.0%	282	0.5306
	Lovastatin	91	42.1%	98	45.4%	27	12.5%	0	0.0%	216	0.8524
	Rosuvastatin	82	41.8%	89	45.4%	25	12.8%	0	0.0%	196	0.9023
**Bisphosphonates**	Alendronate	79	44.1%	82	45.8%	18	10.1%	0	0.0%	179	0.5794
	Risedronate	5	41.7%	6	50.0%	1	8.3%	0	0.0%	12	0.9416
	Ibandronate	4	40.0%	5	50.0%	1	10.0%	0	0.0%	10	0.9634
	Zoledronic acid	1	50.0%	1	50.0%	0	0.0%	0	0.0%	2	1.0000
**Proton Pump Inhibitors**	Omeprazole	214	41.8%	231	45.1%	67	13.1%	0	0.0%	512	0.2982
	Pantoprazole	133	41.6%	144	45.0%	43	13.4%	0	0.0%	320	0.4907
	Lansoprazole	57	42.5%	61	45.5%	16	11.9%	0	0.0%	134	0.9254
	Esomeprazole	42	41.6%	46	45.5%	13	12.9%	0	0.0%	101	0.9238
	Dexilant	2	40.0%	2	40.0%	1	20.0%	0	0.0%	5	0.9818

**Table 4:** Multinomial logistic regression analysis of significant medications on furcation grades.

Comparison	Medication	Odds Ratio (OR)	95% CI Lower	95% CI Upper	*p*-Value
**Grade 2 vs. Grade 1**	Takes Lisinopril	1.15	1.01	1.31	0.038
	Takes Aspirin	1.10	0.95	1.28	0.208
	Takes Atorvastatin	1.13	0.98	1.32	0.101
	Takes Simvastatin	1.10	0.92	1.32	0.288
**Grade 3 vs. Grade 1**	**Takes Lisinopril**	1.50	1.25	1.81	0.000
	**Takes Aspirin**	1.48	1.21	1.82	0.000
	**Takes Atorvastatin**	1.54	1.26	1.89	0.000
	**Takes Simvastatin**	1.46	1.16	1.85	0.001
**Grade 4 vs. Grade 1**	Takes Lisinopril	0.58	0.08	4.38	0.598
	Takes Aspirin	0.73	0.17	3.16	0.669
	Takes Atorvastatin	-	-	-	-
	Takes Simvastatin	-	-	-	-

## Data Availability

The data presented in this study are available upon request from the corresponding author due to privacy/ethical restrictions. The data are not publicly available as they contain information that could compromise the privacy of research participants. The dataset contains sensitive personal information, and participant consent did not include public data sharing.
